# Investigating the GWAS-Implicated Loci for Rheumatoid Arthritis in the Pakistani Population

**DOI:** 10.1155/2020/1910215

**Published:** 2020-07-31

**Authors:** Muhammad Muaaz Aslam, Peter John, Kang-Hsien Fan, Attya Bhatti, Wajahat Aziz, Bashir Ahmed, Eleanor Feingold, F. Yesim Demirci, M. Ilyas Kamboh

**Affiliations:** ^1^Atta-ur-Rahman School of Applied Biosciences (ASAB), National University of Sciences and Technology (NUST), Islamabad, Pakistan; ^2^Department of Human Genetics, Graduate School of Public Health (GSPH), University of Pittsburgh, Pittsburgh, PA, USA; ^3^Department of Rheumatology, Pakistan Institute of Medical Sciences (PIMS), Islamabad, Pakistan

## Abstract

Rheumatoid arthritis (RA) is a complex and multifactorial autoimmune disorder with the involvement of multiple genetic and environmental factors. Genome-wide association studies (GWAS) have identified more than 50 RA genetic loci in European populations. Given the anticipated overlap of RA-relevant genes and pathways across different ethnic groups, we sought to replicate 58 GWAS-implicated SNPs reported in Europeans in Pakistani subjects. 1,959 unrelated subjects comprising 1,222 RA cases and 737 controls were collected from three rheumatology facilities in Pakistan. Genotyping was performed using iPLEX or TaqMan® methods. A total of 50 SNPs were included in the final association analysis after excluding those that failed assay design/run or postrun QC analysis. Fourteen SNPs (*LINC00824*/rs1516971, *PADI4*/rs2240336, *CEP57*/rs4409785, *CTLA4*/rs3087243, *STAT4*/rs13426947, *HLA-B/MICA*/rs2596565, *C5orf30*/rs26232, *CCL21*/rs951005, *GATA3*/rs2275806, *VPS37C*/rs595158, *HLA-DRB1*/rs660895, *EOMES*/rs3806624, *SPRED2*/rs934734, and *RUNX1*/rs9979383) were replicated in our Pakistani sample at false discovery rate (FDR) of <0.20 with nominal *p* values ranging from 4.73*E*-06 to 3.48*E*-02. Our results indicate that several RA susceptibility loci are shared between Pakistani and European populations, supporting the role of common genes/pathways.

## 1. Introduction

Rheumatoid arthritis (RA) is a complex autoimmune disorder due to the involvement of many genetic, environmental, and stochastic elements in its etiology [1, 2]. It is characterized by continuous inflammation associated with altered expression of different proinflammatory (TNF*α*, IL-1, IL-17) and anti-inflammatory (IL-4, IL-10, IL-13, IL-35) cytokines and activation of B and T cells, which leads to the destruction of synovial joints and ultimately physical disability [3, 4]. Worldwide, RA affects 0.5 to 1% of the population [5]. RA prevalence does not differ significantly between rural and urban areas [6]. It can affect both sexes at any age, but data suggest that women are three times more prone than men [7]. There is a significant genetic susceptibility to RA with 50-60% heritability estimates [8]. Twin studies also revealed high concordance rates in monozygotic twins (12.3-15.4%) relative to dizygotic twins (3.5%), strongly indicating the presence of a genetic component in RA [9].

During the last two decades, many genetic studies, including genome-wide association studies (GWAS), have been conducted to understand the genetic basis of RA and this has led to the identification of more than 50 risk loci [10]. Besides *HLA-DRB1*, many non-*HLA* loci have been implicated that are involved in multiple functions/pathways including immune activation (NF-*κ*B signaling, JAK-STAT pathway, regulation, and activation of CD4^+^T-cells, complement activation), fibroblast differentiation and dedifferentiation, and bone modeling and repair [11, 12].

Thus far, the major focus of genetic studies on RA has largely been on Europeans or European-derived populations, with only limited data available on other populations [13], especially in South Asians [14]. In an effort to expand genetic studies on RA in South Asians and to further delineate its genetic basis, we sought to replicate 58 genome-wide significant single nucleotide polymorphisms (SNPs) from 58 loci previously implicated in Europeans or European-derived populations [15–18] in Pakistani population where there is the paucity of such data.

## 2. Material and Methods

### 2.1. Study Subjects

A total of 1,959 subjects (1,222 cases, 737 controls) were included in our study. Blood samples and relevant clinical information were collected from three major public or private rheumatology clinics in Pakistan: Pakistan Institute of medical sciences (PIMS), Military hospital, and Rehmat Noor Clinic. All cases included in this study (mean age ± SD = 43.1 ± 12.33, 78.6% women) were clinically diagnosed by rheumatologists and met the American College of Rheumatology (ACR) 1987 classification criteria for RA [19]. All controls included in this study (mean age ± SD = 40.7 ± 12.49, 39.5% women) were recruited from the general population and were free of any autoimmune disease at the time of recruitment. A screening questionnaire was filled out and a written informed consent was obtained from each subject at the time of the recruitment. All blood samples were collected in EDTA tubes to avoid coagulation and processed shortly after the collection. The study was approved by the Institutional review board (IRB) of the University of Pittsburgh, USA (IRB no. PRO12110472).

### 2.2. Genomic DNA Extraction

Genomic DNA was extracted from whole blood using either the standard phenol-chloroform extraction method or GeneJET Whole Blood Genomic DNA Purification (Thermo Scientific USA) and quantified using the NanoDrop™ 2000 spectrophotometer (Thermo Scientific USA).

### 2.3. Genotyping

A total of 58 RA-associated genome-wide significant SNPs (*p* < 5*E* − 08) was selected from the previously published GWAS in subjects of European ancestry (Table 1). SNP genotyping was performed using either the TaqMan® (Applied Biosystems, Thermo Fisher Scientific) or iPLEX® Gold (Agena Bioscience) methods and following the manufacturer's design/order instructions and protocols. After the thermal cycling of the TaqMan® assays and DNAs on 384-well plates, the endpoint fluorescence reading was performed on a QuantStudio™ 12K Flex system (Applied Biosystems, Thermo Fisher Scientific). The iPLEX® Gold genotyping was performed in the Core laboratories of the University of Pittsburgh. 18% replicates were used to test genotyping consistency.

### 2.4. Statistical Analysis

Concordance to Hardy-Weinberg Equilibrium (HWE) was tested using the Chi-square goodness of fit test. Departure from HWE was considered at arbitrarily *p* < 1*E* − 05. Logistic regression using an additive model and minor allele as the effect allele was employed for case-control association analysis using sex and age as covariates. The Benjamin Hochberg false discovery rate (FDR) was applied to correct for multiple testing [20]. *p* < 0.05 was considered as suggestive evidence of association and FDR (*q* value) of <0.20 as statistically significant as used in previous reports [21, 22]. All analyses were implemented in R, version 3.4.4.

### 2.5. Functional Annotations

To evaluate the potential biological significance of reported genome-wide significant SNPs, we used the Genotype-Tissue Expression (GTEx) database (https://gtexportal.org/home/) to search for expression quantitative trait loci (eQTL) in RA-relevant tissues and whole blood. We also used the RegulomeDB online database (http://regulome.stanford.edu/) to determine possible regulatory functions of the SNPs located in noncoding regions.

## 3. Results

A total of 1,222 unrelated RA cases and 737 controls were recruited for this research study. The prevalence of RA was higher in females (78%) than males (22%), supporting the earlier data that females are more prone to RA [14].

Eight of the 58 genotyped SNPs failed the QC (quality control) during assay design (either iPLEX® Gold/TaqMan® or both). The genotype distribution of all QC-passed 50 SNPs adhered to the HWE. The association analyses results in our Pakistani sample are presented in Table 2. Fourteen SNPs showed nominal significance at *p* < 0.05 and FDR of ≤0.20.

**Figure 1:**
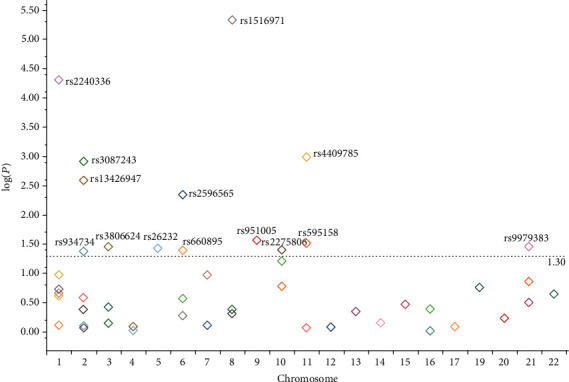
Annotated 50 tested SNPs. SNPs with *p* value < 0.05 are shown above the dotted line.

We found the same direction of association where the minor allele showed the same risk or protective effect as compared to the previous findings on the tested allele (major or minor). In our data, *LINC00824*/rs1516971 showed the most significant association (*p* = 4.73*E* − 06). The second most significant SNP was *PADI4*/rs2240336 (*p* = 5.00*E* − 05) followed by *CEP57*/rs4409785 (*p* = 1.03*E* − 03), *CTLA4*/rs3087243 (*p* = 1.23*E* − 03), *STAT4*/rs13426947 (*p* = 2.59*E* − 03), and *HLA-B-MICA*/rs2596565 (*p* = 4.53*E* − 03). Eight other SNPs showed marginal significance: *C5orf30*/rs26232 (*p* = 3.73*E* − 02), *CCL21*/rs951005 (*p* = 2.75*E* − 02), *GATA3*/rs2275806 (*p* = 4.02*E* − 02), *VPS37C*/rs595158 (*p* = 3.10*E* − 02), *HLA-DRB1*/rs660895 (*p* = 4.02*E* − 02), *EOMES*/rs3806624 (*p* = 3.54*E* − 02), *SPRED2*/rs934734 (*p* = 4.24*E* − 02), and *RUNX1*/rs9979383 (*p* = 3.48*E* − 02).  Figure 1 shows the distribution of tested SNPs across the genome where the SNPs with *p* value < 0.05 are labeled.

Next, we examined the functional significance of all 50 SNPs using the GTEx and RegulomeDB databases.  Table 3 shows the category summaries of RegulomeDB scores, and Table 4 shows 14 SNPs that had a RegulomeDB score of ≤3, indicating strong evidence of potential regulatory role. SNPs falling in this category have more likelihood to affect the binding of transcriptional factors. Out of these fourteen SNPs, only five (*HLA-B/MICA*/rs2596565, *HLA-DRB1*/rs660895, *C5orf30*/rs26232, *CTLA4*/rs3087243, and *RUNX1*/rs9979383) had *p* < 0.05 in our association results. *SYNGR1*/rs909685 had the top RegulomeDB score of 1b followed by *FADS2*/rs968567, *CD40*/rs4810485, *CCR6*/rs3093023, *HLA-B/MICA/*rs2596565, and *HLA-DRB1*/rs660895 with a score of 1f. The latter two SNPs (*HLA-B/MICA/*rs2596565 and *HLA-DRB1*/rs660895) were significant in our sample (*p* = 0.0045 and *p* = 0.0402) and based on RegulomeDB, both SNPs are eQTL for *HLA-DQA1*. While *HLA-DRB1*/rs660895 affects the binding of two proteins (BCLAF1 and POLR2A), no such evidence was found for *HLA-B/MICA/*rs2596565. *SYNGR1*/rs909685 is an intronic variant, which falls in the p53decamer binding motif and affects the binding of five different proteins (MAX, MYC, PAX5, TRIM28, and EBF1). *FADS2*/rs968567 is also an intronic variant and it affects the binding of twenty-eight proteins, including MAX, POLR2A, NFKB1, and NFIC. The binding of these proteins is also affected by the *CD40*/rs4810485 variant. An intronic *CCR6*/rs3093023 variant falls in the Oct-1 binding motif and is eQTL for *CCR6.*

GTEx data eQTL showed the lowest p-eQTL (2.59*E*-34) for *FADS2*/rs968567 that affects *FADS2* gene expression in transformed fibroblast cells; the same variant also affects *TMEM258* (p − eQTL = 3.43*E* − 08), *ZBTB3* (p − eQTL = 0.0168), *MYRF* (p − eQTL = 0.0207), *DAGLA* (p − eQTL = 0.0246), and *RAB3IL1* (p − eQTL = 0.0247). In transformed fibroblast cells, *LINC00824*/rs1516971 was a weak eQTL (*p* = 0.0157) for *RP11-89M16.1.* No data was available for *CTLA*4/rs3087243 in GTEx.

*STAT4*/rs13426947 was eQTL for *STAT4*, *AC005540.3*, and *RP11-647K16.1* in EBV-transformed lymphocyte cells. HLA-B/MICA/rs2596565 was eQTL for multiple immune-related genes including *C4A*, *C4B*, *HLA-S*, *HLA-B*, *HLA-C*, and *MICA* in all three tested tissues. *C5orf30*/rs26232 showed the strongest eQTL (p − eQTL = 1.72*E* − 19) with PPIP5K2 only in whole blood. Additional details of GTEx data are given in supplementary Table S1.

## 4. Discussion

There have been a number of genome-wide association studies on RA over the past decade that have resulted in the identification of many RA susceptibility loci, thus improving our understanding of the complex genetic underpinning of RA [15–17]. Most of the recently published GWAS have been conducted on Europeans and North Americans, which have identified many new RA risk loci and replicated and confirmed the previously reported putative risk loci. Many of these reported risk loci are shared among different ethnic groups, while some are specific to certain populations [23]. Replication studies across different ethnic groups are necessary to better understand the globe-wide population specificity of these established RA risk loci and guide future directions. Since South Asians, including the Pakistani population, have significant European ancestry [24, 25], we chose European originated GWAS-implicated SNPs for replication in Pakistanis. For this purpose, we enrolled 1,959 unrelated RA cases and controls from two public and private Rheumatology facilities, where people from different demographic regions of Pakistan visit for treatment, and we successfully genotyped 50 GWAS—implicated SNPs in those subjects.

In our data set, rs1516971 was the most significant SNP (*p* = 4.73*E* − 06), which is an intronic variant in the *LINC00824* gene at chromosome 8q24.21. Previously, this variant has also been reported in a trans-ethnic association study of RA (*p* = 1.3*E* − 10) [16]. Another SNP (rs6651252) from the same region, which is in complete LD (*r*^2^ = 1.00) with rs1516971, has also been reported to be associated with RA and Crohn's disease [15, 26, 27]. GTEx expression data suggests that rs1516971 affects the expression of *RP11-89M16.1* at chromosome 8 in transformed fibroblast cells (p − eQTL = 0.0157).

The second most significant SNP in our data was rs2240336 (*p* = 5.00*E* − 05), which is present in the 9^th^ intron of *PADI4* at chromosome 1p36. *PADI4* is one of the most important RA susceptibility loci in multiple ethnic groups, including Europeans, Asians, and Latin Americans [28, 29]. Another SNP in this region, rs2301888 that is in strong LD (*r*^2^ = 0.8) with rs2240336, has also been reported to be associated with RA in Europeans and Koreans [30]. However, a study on Iranian population found no significant association of two other SNPs (rs11203367 and rs874881) in the *PADI4* region with RA, which are not in strong LD (*r*^2^ < 0.8) with rs2240336 [31]. GTEx expression data showed that rs2240336 is eQTL for *RP4-798A10.2* (p − eQTL = 0.0191) and *PADI3* (p − eQTL = 0.0203) in EBV transformed lymphocytes. The rs2240336 SNP also controls the expression of *FBXO42* (p − eQTL = 0.00526) in transformed fibroblasts. The third most important SNP in our data is *CEP57*/rs4409785 (p = 1.03E − 03), which is an intergenic variant on chromosome 11q21. This SNP has also been reported to be associated with other immune-related diseases such as multiple sclerosis, vitiligo, and Graves' disease [32–34]. In GTEx expression data, rs4409785 was eQTL for *JRKL* (p − eQTL = 0.037) in EBV-transformed lymphocytes, and for *AP001877.1* (p − eQTL = 0.0188), and *MAML2* (p − eQTL = 0.034) in transformed fibroblast cells.

Among the reported genome-wide significant SNPs that did not show significant association in our sample, the top functional ones based on the GTEx and RegulomeDB database included *SYNGR1*/rs909685, *FADS2*/rs968567, *CD40*/rs4810485, *CCR6*/rs3093023, and *IKZF3*/rs12936409. *SYNGR1*/rs909685 has a RegulomeDB score of 1b and is a strong eQTL for *SYNGR1* in EBV-transformed lymphocytes (p − eQTL = 3.74*E* − 14) and whole blood (p − eQTL = 5.68*E* − 21). *FADS2*/rs968567 has a RegulomeDB score of 1f and is eQTL for *FADS2* in EBV-transformed lymphocytes (p − eQTL = 2.05*E* − 05), whole blood and (p − eQTL = 1.86*E* − 71) transformed fibroblasts (p − eQTL = 2.59*E* − 34). *CD40*/rs4810485 also has a high RegulomeDB score of 1f and is strong eQTL for *CD40* in whole blood (p − eQTL = 1.33*E* − 09) and transformed fibroblasts (p − eQTL = 8.58*E* − 11) but weak eQTL for *CD40* in EBV-transformed lymphocytes (p − eQTL = 0.0272). *CCR6*/rs3093023 with RegulomeDB score of 1f was the strongest eQTL for *RNASET2* in transformed fibroblasts (p − eQTL = 1.08*E* − 16) and whole blood (p − eQTL = 1.31*E* − 15) with no data reported for EBV-transformed lymphocytes in GTEx. *IKZF3*/rs12936409 was a strong eQTL for *ORMDL3* in all three tested tissues. In GTEx expression data, rs12936409 was found to control the expression of *GSDMA*, *GSDMB*, and *ORMDL3* in all tested cells and whole blood.

## 5. Conclusions

We were able to successfully replicate 14 of 50 SNPs selected from previously published GWAS results in European populations in our unique Pakistani population. These findings suggest that there is a sharing of RA risk loci among different population groups. A weakness of our study is the availability of relatively small-sized sample, which may have prevented 36 SNPs from achieving the significance threshold, although they showed a trend for the same directional effects as the reported ones. Further studies using larger samples may help to identify and replicate more RA risk loci in the Pakistani population.

## Figures and Tables

**Table 1:** List of selected GWAS-implicated RA SNPs examined in this study.

SNP	Locus	Chromosomal location (GRCh38)	Variant type	Gene	Reference
rs2228145	1q21	154454494	Missense variant	*IL6R*	[15]
rs2105325	1q25.1	173380586	Intron variant	*LOC100506023*	[16]
rs2843401	1p36	2596694	Intron variant	*MMEL1*	[15]
rs28411352	1p34.3	37812907	3 prime UTR variant	*MTF1*	[16]
rs883220	1p34	38151199	Intron variant	*LOC105378654*	[15]
rs2476601	1p13	113834946	Missense variant	*PTPN22*	[15]
rs6732565	2q13	110850255	Intron variant	*ACOXL*	[16]
rs11676922	2q11	100190478	Intergenic	*AFF3*	[17]
rs6715284	2q33.1	201289674	Intron variant	*ALS2CR12*	[16]
rs3087243	2q33	203874196	Downstream variant	*CTLA4*	[17]
rs34695944	2p16	60897715	Intron variant	*REL*	[15]
rs934734	2p14	65368452	Intron variant	*SPRED2*	[17]
rs13426947	2q32	191068528	Intron variant	*STAT4*	[15]
rs3806624	3p24.1	27723132	Upstream variant	*EOMES*	[16]
rs9826828	3q22.3	136683218	Intron variant	*STAG1*	[16]
rs4452313	3p24.3	17005540	Intron variant	*PLCL2*	[16]
rs13315591	3p14	58571114	Intron variant	*FAM107A*	[17]
rs874040	4p15	26106575	Upstream variant	*RBPJ*	[17]
rs2664035	4p11	48218822	Intron variant	*TEC*	[16]
rs71624119	5q11	56144903	Intron variant	*ANKRD55*	[15]
rs26232	5q21	103261019	Intron variant	*C5orf30*	[17]
rs3093023	6q27	167120802	Intron variant	*CCR6*	[16]
rs2234067	6p21.31	36387877	Upstream variant	*ETV7*	[16]
rs6920220	6q23	137685367	Upstream variant	*TNFAIP3*	[15]
rs2596565	6p21.33	31385552	Upstream variant	*HLA-B/MICA*	[18]
rs6910071	6p21	32315077	Intron variant	*TSBP1*	[17]
rs660895	6p21	32609603	Intron variant	*HLA-DRB1*	[14]
rs4272	7q21.2	92607515	3 prime UTR variant	*CDK6*	[16]
rs10488631	7q32	128954129	Downstream variant	*IRF5*	[17]
rs67250450	7p15.1	28135367	Intron variant	*JAZF1*	[16]
rs678347	8q22.3	101451374	Upstream variant	*GRHL2*	[16]
rs1516971	8q24.21	128529854	Intron variant	*LINC00824*	[16]
rs998731	8q21.13	80183160	Intron variant	*TPD52*	[16]
rs951005	9p13	34743684	Intergenic	*CCL21*	[17]
rs12764378	10q21	62040245	Intron variant	*ARID5B*	[15]
rs2275806	10p14	8053377	Intron variant	*GATA3*	[15]
rs706778	10p15	6056986	Intron variant	*IL2RA*	[17]
rs595158	11q12	61142109	Intron variant	*VPS37C*	[15]
rs4409785	11q21	95578258	Intergenic/unknown	*CEP57*	[16]
rs773125	12q13.2	56001170	Intron variant	*SUOX*	[16]
rs1950897	14q24.1	68293424	Intron variant	*RAD51B*	[16]
rs8043085	15q14	38535939	Intron variant	*RASGRP1*	[15]
rs8026898	15q23	69699078	Intergenic/unknown	*TLE3*	[15]
rs13330176	16q24	85985481	Intergenic/unknown	*IRF8*	[15]
rs4780401	16p13.13	11745470	Upstream variant	*TXNDC11*	[16]
rs12936409	17q12	39887396	Intergenic/unknown	*IKZF3*	[15]
rs34536443	19p13	10352442	Missense variant	*TYK2*	[15]
rs4810485	20q13	46119308	Intron variant	*CD40*	[17]
rs9979383	21q22	35343463	Intron variant	*RUNX1*	[15]
rs1893592	21q22.3	42434957	Intron variant	*UBASH3A*	[16]
rs909685	22q13.1	39351666	Intron variant	*SYNGR1*	[16]
rs2240336	1p36	17347907	Intron variant	*PADI4*	[16]
rs10175798	2p23.1	30226728	Upstream variant	*LBH*	[16]
rs968567	11q12.2	61828092	Intron variant	*FADS2*	[16]
rs10774624	12q24.12	111395984	Upstream variant	*LINC02356*	[16]
rs9603616	13q14.11	39793932	Downstream variant	*COG6*	[16]
rs73194058	21q22.11	33391982	Intergenic/unknown	*IFNGR2*	[16]
rs2834512	21q22	34539301	Intron variant	*RCAN1*	[15]

**Table 2:** Association analysis results for GWAS-implicated RA SNPs in the Pakistani population.

Gene/SNP	Major allele	Minor allele	MAF	Reported GWAS *p* value	Nominal *p* value in Pakistanis	OR (95% CI)	FDR (*q*)
*LINC00824*/rs1516971	T	C	0.099	3.20*E*-11	4.73*E*-06	0.57 (0.45, 0.73)	2.37*E*-04
*PADI4*/rs2240336	C	T	0.452	5.9*E*−9	5.00*E*-05	0.74 (0.64, 0.86)	1.25*E*-03
*CEP57*/rs4409785	T	C	0.262	3.60*E*-09	1.03*E*-03	1.33 (1.12, 1.58)	1.54*E*-02
*CTLA4*/rs3087243	A	G	0.422	1.2*E*−8	1.23*E*-03	1.28 (1.1, 1.49)	1.54*E*-02
*STAT4*/rs13426947	G	A	0.238	7.2*E*−10	2.59*E*-03	1.31 (1.1, 1.56)	2.59*E*-02
*HLA-B/MICA*/rs2596565	G	A	0.119	9.26*E*-09	4.53*E*-03	1.4 (1.11, 1.77)	3.77*E*-02
*C5orf30*/rs26232	C	T	0.181	4.10*E*-08	3.73*E*-02	0.83 (0.69, 0.99)	1.52*E*-01
*CCL21*/rs951005	A	G	0.248	3.90*E*-10	2.75*E*-02	0.82 (0.69, 0.98)	1.52*E*-01
*GATA3*/rs2275806	A	G	0.397	4.6*E*−8	4.02*E*-02	1.17 (1.01, 1.36)	1.52*E*-01
*VPS37C*/rs595158	A	C	0.320	3.4*E*−8	3.10*E*-02	1.19 (1.02, 1.38)	1.52*E*-01
*HLA-DRB1*/rs660895	A	G	0.111	<1*E*-300	4.02*E*-02	1.27 (1.01, 1.59)	1.52*E*-01
*EOMES*/rs3806624	G	A	0.262	2.80*E*-08	3.54*E*-02	0.84 (0.71, 0.99)	1.52*E*-01
*SPRED2*/rs934734	A	G	0.406	5.30*E*-10	4.24*E*-02	1.17 (1.01, 1.35)	1.52*E*-01
*RUNX1*/rs9979383	T	C	0.300	5.0*E*−10	3.48*E*-02	0.84 (0.72, 0.99)	1.52*E*-01
*IL2RA*/rs706778	T	C	0.458	1.40*E*-11	6.21*E*-02	0.87 (0.75, 1.01)	2.07*E*-01
*LOC105378654*/rs883220	C	A	0.212	2.1*E*−8	1.08*E*-01	0.87 (0.73, 1.03)	3.17*E*-01
*IRF5*/rs10488631	T	C	0.196	4.20*E*-11	1.07*E*-01	1.16 (0.97, 1.39)	3.17*E*-01
*RCAN1*/rs2834512	G	A	0.160	2.1*E*−8	1.39*E*-01	0.86 (0.71, 1.05)	3.86*E*-01
*ARID5B*/rs12764378	G	A	0.241	4.5*E*−10	1.64*E*-01	1.13 (0.95, 1.34)	4.33*E*-01
*TYK2*/rs34536443	G	C	0.010	2.3*E*−14	1.73*E*-01	0.62 (0.31, 1.23)	4.33*E*-01
*IL6R*/rs2228145	A	C	0.323	1.3*E*−8	1.89*E*-01	0.9 (0.77, 1.05)	4.50*E*-01
*PTPN22*/rs2476601	G	A	0.016	7.5*E*−77	2.21*E*-01	1.47 (0.79, 2.73)	4.93*E*-01
*SYNGR1*/rs909685	T	A	0.480	6.40*E*-12	2.27*E*-01	1.09 (0.95, 1.26)	4.93*E*-01
*MTF1*/rs28411352	C	T	0.222	5.90*E*-09	2.42*E*-01	1.11 (0.93, 1.32)	5.05*E*-01
*CCR6*/rs3093023	G	A	0.425	1.50*E*-11	2.70*E*-01	1.09 (0.94, 1.26)	5.19*E*-01
*ALS2CR12*/rs6715284	C	G	0.185	2.50*E*-09	2.61*E*-01	1.11 (0.92, 1.35)	5.19*E*-01
*UBASH3A*/rs1893592	A	C	0.241	9.80*E*-09	3.11*E*-01	0.92 (0.77, 1.08)	5.75*E*-01
*IRF8*/rs13330176	T	A	0.243	4.0*E*−8	4.04*E*-01	0.93 (0.78, 1.11)	6.13*E*-01
*PLCL2*/rs4452313	A	T	0.492	5.2*E*-11	3.76*E*-01	1.07 (0.92, 1.24)	6.13*E*-01
*ACOXL*/rs6732565	G	A	0.444	9.40*E*-09	4.15*E*-01	0.94 (0.81, 1.09)	6.13*E*-01
*GRHL2*/rs678347	A	G	0.425	7.30*E*-10	4.17*E*-01	1.06 (0.92, 1.23)	6.13*E*-01
*TLE3*/rs8026898	G	A	0.303	9.16.*E*-14	3.50*E*-01	0.93 (0.79, 1.09)	6.13*E*-01
*RASGRP1*/rs8043085	G	T	0.280	1.4*E*−10	3.64*E*-01	1.08 (0.92, 1.27)	6.13*E*-01
*REL*/rs34695944	T	C	0.112	1.4*E*−10	4.05*E*-01	1.1 (0.88, 1.39)	6.13*E*-01
*COG6*/rs9603616	C	T	0.260	2.80*E*-11	4.47*E*-01	0.94 (0.8, 1.11)	6.38*E*-01
*TPD52*/rs998731	T	C	0.434	6.60*E*-09	5.04*E*-01	0.95 (0.82, 1.1)	7.00*E*-01
*TNFAIP3*/rs6920220	G	A	0.132	2.3*E*−13	5.33*E*-01	1.07 (0.86, 1.34)	7.20*E*-01
*CD40*/rs4810485	G	T	0.250	2.8*E*−9	6.03*E*-01	0.96 (0.81, 1.13)	7.94*E*-01
*RAD51B*/rs1950897	T	C	0.208	5.00*E*-08	7.02*E*-01	1.04 (0.86, 1.24)	8.98*E*-01
*FAM107A*/rs13315591	T	C	0.043	4.60*E*-08	7.25*E*-01	1.06 (0.75, 1.51)	8.98*E*-01
*MMEL1*/rs2843401	C	T	0.461	6.6*E*−9	7.80*E*-01	1.02 (0.88, 1.18)	8.98*E*-01
*RBPJ*/rs874040	G	C	0.159	1.00*E*-16	8.20*E*-01	0.98 (0.81, 1.18)	8.98*E*-01
*IKZF3*/rs12936409	C	T	0.392	2.8*E*−9	8.19*E*-01	0.98 (0.85, 1.14)	8.98*E*-01
*LBH*/rs10175798	A	G	0.474	4.20*E*-08	8.46*E*-01	1.01 (0.88, 1.17)	8.98*E*-01
*LINC02356*/rs10774624	A	G	0.138	6.90*E*-09	8.38*E*-01	0.98 (0.79, 1.2)	8.98*E*-01
*FADS2*/rs968567	G	A	0.084	1.80*E*-08	8.62*E*-01	0.98 (0.75, 1.27)	8.98*E*-01
*AFF3*/rs11676922	T	A	0.485	1.00*E*-14	7.91*E*-01	0.98 (0.85, 1.14)	8.98*E*-01
*CDK6*/rs4272	A	G	0.083	1.2*E*-8	7.64*E*-01	0.96 (0.74, 1.24)	8.98*E*-01
*TEC*/rs2664035	G	A	0.256	3.30*E*-08	9.54*E*-01	1 (0.84, 1.17)	9.70*E*-01
*TXNDC11*/rs4780401	T	G	0.430	8.70*E*-09	9.70*E*-01	1 (0.86, 1.16)	9.70*E*-01

**Table 3:** RegulomeDB score description.

Score	Description
Likely to affect binding and linked to the expression of a gene target	
1a	eQTL + TF binding + matched TF motif + matched DNase footprint + DNase peak
1b	eQTL + TF binding + any motif + DNase footprint + DNase peak
1c	eQTL + TF binding + matched TF motif + DNase peak
1d	eQTL + TF binding + any motif + DNase peak
1e	eQTL + TF binding + matched TF motif
1f	eQTL + TF binding/DNase peak
Likely to affect binding	
2a	TF binding + matched TF motif + matched DNase footprint + DNase peak
2b	TF binding + any motif + DNase footprint + DNase peak
2c	TF binding + matched TF motif + DNase peak
Less likely to affect binding	
3a	TF binding + any motif + DNase peak
3b	TF binding + matched TF motif
Minimal binding evidence	
4	TF binding + DNase peak
5	TF binding or DNase peak
6	Motif hit
7	No data available

**Table 4:** Details of study SNPs (RegulomeDB Score ≤ 3) with putative regulatory functions.

SNP	Chr.	Score	eQTL	Bound protein	Motifs
*SYNGR1*/rs909685	chr22	1b	SYNGR1	MAX, MYC, PAX5, TRIM28, EBF1	p53decamer
*FADS2*/rs968567	chr11	1f	NXF1	CTCF, E2F4, EP300, ETS1, FOSL2, FOXP2, GABPA, GABPB1, GATA1, HNF4A, HNF4G, MAX, MYBL2, NFIC, NFKB1, NFYB, PML, POLR2A, RAD21, REST, SIN3A, SP1, SREBF1, SREBF2, TAF1, TCF12, YY1, ZBTB7A	
*CD40*/rs4810485	chr20	1f	CD40	POLR2A, NFKB1, NFIC, MEF2C, MEF2A, IRF1, IKZF1, FOXM1, BCL3, BATF	
*CCR6*/rs3093023	chr6	1f	CCR6		Oct_1
*HLAB*/*MICA*/rs2596565	chr6	1f	BTN3A2, HLA-A, HLA-C, HLA-DPB1, HLA-DQA1, HLA-DQB1, HLA-DRB1, HLA-G, HLA-H, VARSL		
*HLA-DRB1*/rs660895	chr6	1f	HLA-DQA2, HLA-DQA1	BCLAF1, POLR2A	
*GRHL2*/rs678347	chr8	2a		YY1, CEBPB, EP300, FOXA2, JUND, STAT3, TCF12, USF2, SIN3A	HNF3, Foxa2, FOXF2, Freac-2, Freac-4, FOXA1, HNF3, FOXC1
*PTPN22*/rs2476601	chr1	2b		FOS, STAT3	Gabpa, Etv1, Elk1, Erg, Ehf, PU.1
*LBH*/rs10175798	chr2	2b		ATF2, FOXM1, RUNX3, NFIC, SPI1, MEF2A, BATF	STAT1:STAT1
*RBPJ*/rs874040	chr4	2b		GATA2, PML, TAL1	MEF-2
*C5orf30*/rs26232	chr5	2b		BHLHE40, CHD1, EP300, FOXM1, MAZ, NFATC1, NFIC, RUNX3, TBL1XR1, TBP, USF2, ATF2, MEF2A, MEF2C, NFKB1	ICSBP
*RUNX1*/rs9979383	chr21	3a		MYC, NFKB1	E47, FIGLA, ID4, MESP1, SNAI2
*CTLA4*/rs3087243	chr2	3a		MAX, FOS, STAT3	Bbx
*IRF5*/rs10488631	chr7	3a		GATA2	Nanog

## Data Availability

The data used to support the findings of this study are included within the article.

## Supplementary Materials

Supplementary Materials S1: additional details of GTEx data.
