# Supplementary material for: Investigating the GWAS-Implicated Loci for Rheumatoid Arthritis in the Pakistani Population
Source: Dis Markers. 2020 Jul 31;2020:1910215. doi: 10.1155/2020/1910215 (PMC7422001; doi:10.1155/2020/1910215)
Supplement: Supplementary Materials — S1: additional details of GTEx data. [file 1910215.f1.docx]

S1: Additional details of GTEx data

| SNP | Gene | Chr. | BP | A1 | A2 | p_eQTL | Tissue |
| --- | --- | --- | --- | --- | --- | --- | --- |
| rs12936409 | ORMDL3 | 17 | 38043649 | T | C | 4.80E-16 | Cells_EBV-transformed_lymphocytes |
| rs909685 | SYNGR1 | 22 | 39747671 | A | T | 3.74E-14 | Cells_EBV-transformed_lymphocytes |
| rs660895 | HLA-DQA2 | 6 | 32577380 | G | A | 2.72E-13 | Cells_EBV-transformed_lymphocytes |
| rs2596565 | C4A | 6 | 31353329 | A | G | 1.09E-09 | Cells_EBV-transformed_lymphocytes |
| rs660895 | HLA-DQA1 | 6 | 32577380 | G | A | 1.28E-08 | Cells_EBV-transformed_lymphocytes |
| rs660895 | HLA-DQB2 | 6 | 32577380 | G | A | 3.14E-08 | Cells_EBV-transformed_lymphocytes |
| rs12936409 | GSDMB | 17 | 38043649 | T | C | 5.82E-07 | Cells_EBV-transformed_lymphocytes |
| rs12936409 | RP11-94L15.2 | 17 | 38043649 | T | C | 5.03E-06 | Cells_EBV-transformed_lymphocytes |
| rs660895 | HLA-DQB1 | 6 | 32577380 | G | A | 6.89E-06 | Cells_EBV-transformed_lymphocytes |
| rs968567 | FADS2 | 11 | 61595564 | T | C | 2.05E-05 | Cells_EBV-transformed_lymphocytes |
| rs595158 | VPS37C | 11 | 60909581 | A | C | 7.98E-05 | Cells_EBV-transformed_lymphocytes |
| rs26232 | PPIP5K2 | 5 | 102596720 | T | C | 0.000104 | Cells_EBV-transformed_lymphocytes |
| rs6715284 | PPIL3 | 2 | 202154397 | G | C | 0.000108 | Cells_EBV-transformed_lymphocytes |
| rs2596565 | C4B | 6 | 31353329 | A | G | 0.000142 | Cells_EBV-transformed_lymphocytes |
| rs968567 | TMEM258 | 11 | 61595564 | T | C | 0.000301 | Cells_EBV-transformed_lymphocytes |
| rs2596565 | C6orf48 | 6 | 31353329 | A | G | 0.000525 | Cells_EBV-transformed_lymphocytes |
| rs8043085 | RASGRP1 | 15 | 38828140 | T | G | 0.00108 | Cells_EBV-transformed_lymphocytes |
| rs28411352 | POU3F1 | 1 | 38278579 | T | C | 0.00127 | Cells_EBV-transformed_lymphocytes |
| rs2476601 | BCL2L15 | 1 | 114377568 | G | A | 0.0014 | Cells_EBV-transformed_lymphocytes |
| rs2596565 | VARS2 | 6 | 31353329 | A | G | 0.00145 | Cells_EBV-transformed_lymphocytes |
| rs2596565 | HCG22 | 6 | 31353329 | A | G | 0.00164 | Cells_EBV-transformed_lymphocytes |
| rs909685 | APOBEC3H | 22 | 39747671 | A | T | 0.00174 | Cells_EBV-transformed_lymphocytes |
| rs2596565 | HLA-S | 6 | 31353329 | A | G | 0.00202 | Cells_EBV-transformed_lymphocytes |
| rs12936409 | GSDMA | 17 | 38043649 | T | C | 0.00202 | Cells_EBV-transformed_lymphocytes |
| rs12936409 | ZPBP2 | 17 | 38043649 | T | C | 0.00232 | Cells_EBV-transformed_lymphocytes |
| rs951005 | GALT | 9 | 34743681 | A | G | 0.0031 | Cells_EBV-transformed_lymphocytes |
| rs2596565 | RNF5 | 6 | 31353329 | A | G | 0.00322 | Cells_EBV-transformed_lymphocytes |
| rs660895 | NOTCH4 | 6 | 32577380 | G | A | 0.00347 | Cells_EBV-transformed_lymphocytes |
| rs951005 | TESK1 | 9 | 34743681 | A | G | 0.00371 | Cells_EBV-transformed_lymphocytes |
| rs660895 | C4A | 6 | 32577380 | G | A | 0.00399 | Cells_EBV-transformed_lymphocytes |
| rs2843401 | TNFRSF14 | 1 | 2528133 | C | T | 0.00444 | Cells_EBV-transformed_lymphocytes |
| rs13426947 | STAT4 | 2 | 191933254 | A | G | 0.0049 | Cells_EBV-transformed_lymphocytes |
| rs28411352 | UTP11L | 1 | 38278579 | T | C | 0.00522 | Cells_EBV-transformed_lymphocytes |
| rs12936409 | IKZF3 | 17 | 38043649 | T | C | 0.00527 | Cells_EBV-transformed_lymphocytes |
| rs13426947 | RP11-647K16.1 | 2 | 191933254 | A | G | 0.00563 | Cells_EBV-transformed_lymphocytes |
| rs968567 | DDB1 | 11 | 61595564 | T | C | 0.00597 | Cells_EBV-transformed_lymphocytes |
| rs12936409 | MED24 | 17 | 38043649 | T | C | 0.00636 | Cells_EBV-transformed_lymphocytes |
| rs660895 | RNF5 | 6 | 32577380 | G | A | 0.00649 | Cells_EBV-transformed_lymphocytes |
| rs2843401 | RP3-395M20.8 | 1 | 2528133 | C | T | 0.00712 | Cells_EBV-transformed_lymphocytes |
| rs2596565 | LINC00243 | 6 | 31353329 | A | G | 0.00778 | Cells_EBV-transformed_lymphocytes |
| rs2664035 | TEC | 4 | 48220839 | A | G | 0.00785 | Cells_EBV-transformed_lymphocytes |
| rs8043085 | RP11-102L12.2 | 15 | 38828140 | T | G | 0.00827 | Cells_EBV-transformed_lymphocytes |
| rs2843401 | RP3-395M20.7 | 1 | 2528133 | C | T | 0.00952 | Cells_EBV-transformed_lymphocytes |
| rs2596565 | HLA-C | 6 | 31353329 | A | G | 0.0098 | Cells_EBV-transformed_lymphocytes |
| rs678347 | KB-1615E4.3 | 8 | 102463602 | A | G | 0.00997 | Cells_EBV-transformed_lymphocytes |
| rs28411352 | INPP5B | 1 | 38278579 | T | C | 0.0115 | Cells_EBV-transformed_lymphocytes |
| rs2596565 | HCP5 | 6 | 31353329 | A | G | 0.0124 | Cells_EBV-transformed_lymphocytes |
| rs909685 | RP3-508I15.21 | 22 | 39747671 | A | T | 0.0127 | Cells_EBV-transformed_lymphocytes |
| rs28411352 | FHL3 | 1 | 38278579 | T | C | 0.0131 | Cells_EBV-transformed_lymphocytes |
| rs706778 | PFKFB3 | 10 | 6098949 | T | C | 0.0135 | Cells_EBV-transformed_lymphocytes |
| rs2596565 | PPT2 | 6 | 31353329 | A | G | 0.0137 | Cells_EBV-transformed_lymphocytes |
| rs1950897 | ZFYVE26 | 14 | 68760141 | T | C | 0.0138 | Cells_EBV-transformed_lymphocytes |
| rs2596565 | NELFE | 6 | 31353329 | A | G | 0.014 | Cells_EBV-transformed_lymphocytes |
| rs595158 | DDB1 | 11 | 60909581 | A | C | 0.0143 | Cells_EBV-transformed_lymphocytes |
| rs4780401 | SNN | 16 | 11839326 | T | G | 0.0145 | Cells_EBV-transformed_lymphocytes |
| rs968567 | EML3 | 11 | 61595564 | T | C | 0.0145 | Cells_EBV-transformed_lymphocytes |
| rs660895 | HLA-DRB6 | 6 | 32577380 | G | A | 0.0153 | Cells_EBV-transformed_lymphocytes |
| rs4780401 | RP11-490O6.2 | 16 | 11839326 | T | G | 0.0154 | Cells_EBV-transformed_lymphocytes |
| rs10488631 | RP11-155G14.5 | 7 | 128594183 | C | T | 0.0159 | Cells_EBV-transformed_lymphocytes |
| rs660895 | HCG25 | 6 | 32577380 | G | A | 0.0161 | Cells_EBV-transformed_lymphocytes |
| rs883220 | C1orf109 | 1 | 38616871 | A | C | 0.0162 | Cells_EBV-transformed_lymphocytes |
| rs968567 | SLC15A3 | 11 | 61595564 | T | C | 0.0171 | Cells_EBV-transformed_lymphocytes |
| rs2476601 | DCLRE1B | 1 | 114377568 | G | A | 0.0172 | Cells_EBV-transformed_lymphocytes |
| rs660895 | B3GALT4 | 6 | 32577380 | G | A | 0.0172 | Cells_EBV-transformed_lymphocytes |
| rs909685 | PDGFB | 22 | 39747671 | A | T | 0.0187 | Cells_EBV-transformed_lymphocytes |
| rs2240336 | RP4-798A10.2 | 1 | 17674402 | T | C | 0.0191 | Cells_EBV-transformed_lymphocytes |
| rs2843401 | RP3-395M20.12 | 1 | 2528133 | C | T | 0.0194 | Cells_EBV-transformed_lymphocytes |
| rs2240336 | PADI3 | 1 | 17674402 | T | C | 0.0203 | Cells_EBV-transformed_lymphocytes |
| rs2596565 | ABCF1 | 6 | 31353329 | A | G | 0.0205 | Cells_EBV-transformed_lymphocytes |
| rs951005 | UNC13B | 9 | 34743681 | A | G | 0.0208 | Cells_EBV-transformed_lymphocytes |
| rs6920220 | KIAA1244 | 6 | 138006504 | A | G | 0.021 | Cells_EBV-transformed_lymphocytes |
| rs998731 | MRPS28 | 8 | 81095395 | T | C | 0.0213 | Cells_EBV-transformed_lymphocytes |
| rs874040 | MTND4P9 | 4 | 26108197 | C | G | 0.022 | Cells_EBV-transformed_lymphocytes |
| rs13426947 | AC005540.3 | 2 | 191933254 | A | G | 0.0227 | Cells_EBV-transformed_lymphocytes |
| rs2596565 | SKIV2L | 6 | 31353329 | A | G | 0.0229 | Cells_EBV-transformed_lymphocytes |
| rs26232 | PAM | 5 | 102596720 | T | C | 0.0231 | Cells_EBV-transformed_lymphocytes |
| rs2228145 | RP11-263K19.6 | 1 | 154426970 | C | A | 0.0231 | Cells_EBV-transformed_lymphocytes |
| rs34695944 | FAM161A | 2 | 61124850 | C | T | 0.0235 | Cells_EBV-transformed_lymphocytes |
| rs678347 | NCALD | 8 | 102463602 | A | G | 0.0244 | Cells_EBV-transformed_lymphocytes |
| rs998731 | TPD52 | 8 | 81095395 | T | C | 0.0246 | Cells_EBV-transformed_lymphocytes |
| rs660895 | C2 | 6 | 32577380 | G | A | 0.0252 | Cells_EBV-transformed_lymphocytes |
| rs34536443 | TMED1 | 19 | 10463118 | C | G | 0.0257 | Cells_EBV-transformed_lymphocytes |
| rs2228145 | RN7SL431P | 1 | 154426970 | C | A | 0.0257 | Cells_EBV-transformed_lymphocytes |
| rs10488631 | CICP14 | 7 | 128594183 | C | T | 0.0263 | Cells_EBV-transformed_lymphocytes |
| rs2596565 | HLA-B | 6 | 31353329 | A | G | 0.0265 | Cells_EBV-transformed_lymphocytes |
| rs4810485 | CD40 | 20 | 44747947 | G | T | 0.0272 | Cells_EBV-transformed_lymphocytes |
| rs13330176 | RP11-463O9.9 | 16 | 86019087 | A | T | 0.0274 | Cells_EBV-transformed_lymphocytes |
| rs6732565 | LINC00116 | 2 | 111607832 | G | A | 0.0279 | Cells_EBV-transformed_lymphocytes |
| rs2596565 | EGFL8 | 6 | 31353329 | A | G | 0.0287 | Cells_EBV-transformed_lymphocytes |
| rs6715284 | AC007283.4 | 2 | 202154397 | G | C | 0.0288 | Cells_EBV-transformed_lymphocytes |
| rs2596565 | CYP21A1P | 6 | 31353329 | A | G | 0.0292 | Cells_EBV-transformed_lymphocytes |
| rs28411352 | C1orf122 | 1 | 38278579 | T | C | 0.0295 | Cells_EBV-transformed_lymphocytes |
| rs11676922 | AFF3 | 2 | 100806940 | A | T | 0.03 | Cells_EBV-transformed_lymphocytes |
| rs2228145 | RP11-216N14.7 | 1 | 154426970 | C | A | 0.0301 | Cells_EBV-transformed_lymphocytes |
| rs660895 | PRRT1 | 6 | 32577380 | G | A | 0.0302 | Cells_EBV-transformed_lymphocytes |
| rs2596565 | XXbac-BPG248L24.12 | 6 | 31353329 | A | G | 0.0313 | Cells_EBV-transformed_lymphocytes |
| rs28411352 | ZC3H12A | 1 | 38278579 | T | C | 0.0326 | Cells_EBV-transformed_lymphocytes |
| rs2275806 | TAF3 | 10 | 8095340 | A | G | 0.0329 | Cells_EBV-transformed_lymphocytes |
| rs2228145 | PBXIP1 | 1 | 154426970 | C | A | 0.033 | Cells_EBV-transformed_lymphocytes |
| rs998731 | RP11-48B3.4 | 8 | 81095395 | T | C | 0.0331 | Cells_EBV-transformed_lymphocytes |
| rs1893592 | PRDM15 | 21 | 43855067 | C | A | 0.0344 | Cells_EBV-transformed_lymphocytes |
| rs2596565 | PPP1R10 | 6 | 31353329 | A | G | 0.036 | Cells_EBV-transformed_lymphocytes |
| rs998731 | RP11-1149M10.2 | 8 | 81095395 | T | C | 0.0361 | Cells_EBV-transformed_lymphocytes |
| rs4810485 | RPL13P2 | 20 | 44747947 | G | T | 0.0365 | Cells_EBV-transformed_lymphocytes |
| rs4409785 | JRKL | 11 | 95311422 | C | T | 0.037 | Cells_EBV-transformed_lymphocytes |
| rs660895 | CUTA | 6 | 32577380 | G | A | 0.038 | Cells_EBV-transformed_lymphocytes |
| rs2843401 | TTC34 | 1 | 2528133 | C | T | 0.0395 | Cells_EBV-transformed_lymphocytes |
| rs951005 | SIGMAR1 | 9 | 34743681 | A | G | 0.0398 | Cells_EBV-transformed_lymphocytes |
| rs2664035 | TXK | 4 | 48220839 | A | G | 0.0407 | Cells_EBV-transformed_lymphocytes |
| rs4780401 | TXNDC11 | 16 | 11839326 | T | G | 0.0413 | Cells_EBV-transformed_lymphocytes |
| rs951005 | C9orf131 | 9 | 34743681 | A | G | 0.0414 | Cells_EBV-transformed_lymphocytes |
| rs3806624 | CMC1 | 3 | 27764623 | G | A | 0.0428 | Cells_EBV-transformed_lymphocytes |
| rs706778 | DKFZP667F0711 | 10 | 6098949 | T | C | 0.0431 | Cells_EBV-transformed_lymphocytes |
| rs909685 | RPS19BP1 | 22 | 39747671 | A | T | 0.044 | Cells_EBV-transformed_lymphocytes |
| rs2596565 | MICA | 6 | 31353329 | A | G | 0.0466 | Cells_EBV-transformed_lymphocytes |
| rs34536443 | CARM1 | 19 | 10463118 | C | G | 0.0471 | Cells_EBV-transformed_lymphocytes |
| rs11676922 | REV1 | 2 | 100806940 | A | T | 0.0485 | Cells_EBV-transformed_lymphocytes |
| rs10488631 | RP11-274B21.4 | 7 | 128594183 | C | T | 0.0489 | Cells_EBV-transformed_lymphocytes |
| rs2834512 | GART | 21 | 35911599 | A | G | 0.0491 | Cells_EBV-transformed_lymphocytes |
| rs10488631 | AC018638.1 | 7 | 128594183 | C | T | 0.0491 | Cells_EBV-transformed_lymphocytes |
| rs968567 | FADS2 | 11 | 61595564 | T | C | 2.59E-34 | Cells_Transformed_fibroblasts |
| rs3093023 | RNASET2 | 6 | 167534290 | A | G | 1.08E-16 | Cells_Transformed_fibroblasts |
| rs2596565 | C4B | 6 | 31353329 | A | G | 3.22E-16 | Cells_Transformed_fibroblasts |
| rs6715284 | PPIL3 | 2 | 202154397 | G | C | 1.53E-15 | Cells_Transformed_fibroblasts |
| rs9603616 | COG6 | 13 | 40368069 | T | C | 2.90E-14 | Cells_Transformed_fibroblasts |
| rs2596565 | HLA-C | 6 | 31353329 | A | G | 2.16E-11 | Cells_Transformed_fibroblasts |
| rs4810485 | CD40 | 20 | 44747947 | G | T | 8.58E-11 | Cells_Transformed_fibroblasts |
| rs2596565 | C4A | 6 | 31353329 | A | G | 2.82E-10 | Cells_Transformed_fibroblasts |
| rs968567 | TMEM258 | 11 | 61595564 | T | C | 3.43E-08 | Cells_Transformed_fibroblasts |
| rs34695944 | PUS10 | 2 | 61124850 | C | T | 9.79E-08 | Cells_Transformed_fibroblasts |
| rs3093023 | AL133458.1 | 6 | 167534290 | A | G | 1.12E-07 | Cells_Transformed_fibroblasts |
| rs660895 | HLA-DOB | 6 | 32577380 | G | A | 2.32E-07 | Cells_Transformed_fibroblasts |
| rs12936409 | PSMD3 | 17 | 38043649 | T | C | 6.31E-07 | Cells_Transformed_fibroblasts |
| rs4272 | GATAD1 | 7 | 92236829 | G | A | 6.24E-06 | Cells_Transformed_fibroblasts |
| rs2596565 | HCG20 | 6 | 31353329 | A | G | 6.81E-06 | Cells_Transformed_fibroblasts |
| rs28411352 | INPP5B | 1 | 38278579 | T | C | 1.09E-05 | Cells_Transformed_fibroblasts |
| rs2596565 | LY6G5B | 6 | 31353329 | A | G | 1.17E-05 | Cells_Transformed_fibroblasts |
| rs951005 | RP11-195F19.9 | 9 | 34743681 | A | G | 6.54E-05 | Cells_Transformed_fibroblasts |
| rs2596565 | VARS2 | 6 | 31353329 | A | G | 0.000107 | Cells_Transformed_fibroblasts |
| rs2476601 | AP4B1-AS1 | 1 | 114377568 | G | A | 0.000124 | Cells_Transformed_fibroblasts |
| rs2596565 | MICB | 6 | 31353329 | A | G | 0.000169 | Cells_Transformed_fibroblasts |
| rs2843401 | FAM213B | 1 | 2528133 | C | T | 0.000174 | Cells_Transformed_fibroblasts |
| rs12936409 | ORMDL3 | 17 | 38043649 | T | C | 0.00018 | Cells_Transformed_fibroblasts |
| rs1950897 | TMEM229B | 14 | 68760141 | T | C | 0.000241 | Cells_Transformed_fibroblasts |
| rs2596565 | PSORS1C1 | 6 | 31353329 | A | G | 0.000378 | Cells_Transformed_fibroblasts |
| rs2596565 | PPP1R18 | 6 | 31353329 | A | G | 0.000457 | Cells_Transformed_fibroblasts |
| rs12936409 | MSL1 | 17 | 38043649 | T | C | 0.000461 | Cells_Transformed_fibroblasts |
| rs2843401 | RP3-395M20.8 | 1 | 2528133 | C | T | 0.000589 | Cells_Transformed_fibroblasts |
| rs9979383 | KCNE2 | 21 | 36715761 | T | C | 0.000721 | Cells_Transformed_fibroblasts |
| rs2596565 | CYP21A1P | 6 | 31353329 | A | G | 0.000781 | Cells_Transformed_fibroblasts |
| rs660895 | XXbac-BPG154L12.4 | 6 | 32577380 | G | A | 0.000785 | Cells_Transformed_fibroblasts |
| rs12936409 | STARD3 | 17 | 38043649 | T | C | 0.00115 | Cells_Transformed_fibroblasts |
| rs6715284 | SGOL2 | 2 | 202154397 | G | C | 0.00124 | Cells_Transformed_fibroblasts |
| rs934734 | AC074391.1 | 2 | 65595586 | A | G | 0.00132 | Cells_Transformed_fibroblasts |
| rs6715284 | RNU6-312P | 2 | 202154397 | G | C | 0.00161 | Cells_Transformed_fibroblasts |
| rs2476601 | PHTF1 | 1 | 114377568 | G | A | 0.00177 | Cells_Transformed_fibroblasts |
| rs2596565 | LINC00243 | 6 | 31353329 | A | G | 0.00187 | Cells_Transformed_fibroblasts |
| rs2596565 | ATP6V1G2 | 6 | 31353329 | A | G | 0.00212 | Cells_Transformed_fibroblasts |
| rs706778 | RBM17 | 10 | 6098949 | T | C | 0.00267 | Cells_Transformed_fibroblasts |
| rs2228145 | S100A13 | 1 | 154426970 | C | A | 0.0029 | Cells_Transformed_fibroblasts |
| rs2596565 | MICA | 6 | 31353329 | A | G | 0.00296 | Cells_Transformed_fibroblasts |
| rs12936409 | ERBB2 | 17 | 38043649 | T | C | 0.00301 | Cells_Transformed_fibroblasts |
| rs1950897 | DDX18P1 | 14 | 68760141 | T | C | 0.00305 | Cells_Transformed_fibroblasts |
| rs34536443 | ICAM5 | 19 | 10463118 | C | G | 0.00307 | Cells_Transformed_fibroblasts |
| rs3093023 | RP1-167A14.2 | 6 | 167534290 | A | G | 0.00364 | Cells_Transformed_fibroblasts |
| rs4780401 | TXNDC11 | 16 | 11839326 | T | G | 0.00369 | Cells_Transformed_fibroblasts |
| rs6715284 | CASP8 | 2 | 202154397 | G | C | 0.00392 | Cells_Transformed_fibroblasts |
| rs660895 | PSMB8 | 6 | 32577380 | G | A | 0.00438 | Cells_Transformed_fibroblasts |
| rs951005 | UBAP2 | 9 | 34743681 | A | G | 0.00522 | Cells_Transformed_fibroblasts |
| rs6715284 | KCTD18 | 2 | 202154397 | G | C | 0.00524 | Cells_Transformed_fibroblasts |
| rs2240336 | FBXO42 | 1 | 17674402 | T | C | 0.00526 | Cells_Transformed_fibroblasts |
| rs660895 | LY6G5B | 6 | 32577380 | G | A | 0.00599 | Cells_Transformed_fibroblasts |
| rs6920220 | RP11-356I2.4 | 6 | 138006504 | A | G | 0.00615 | Cells_Transformed_fibroblasts |
| rs34536443 | RPL10P15 | 19 | 10463118 | C | G | 0.00712 | Cells_Transformed_fibroblasts |
| rs2228145 | RP11-263K19.6 | 1 | 154426970 | C | A | 0.00743 | Cells_Transformed_fibroblasts |
| rs26232 | NUDT12 | 5 | 102596720 | T | C | 0.00845 | Cells_Transformed_fibroblasts |
| rs2664035 | TEC | 4 | 48220839 | A | G | 0.00883 | Cells_Transformed_fibroblasts |
| rs660895 | TAP2 | 6 | 32577380 | G | A | 0.00984 | Cells_Transformed_fibroblasts |
| rs660895 | HLA-DRA | 6 | 32577380 | G | A | 0.0103 | Cells_Transformed_fibroblasts |
| rs28411352 | SF3A3 | 1 | 38278579 | T | C | 0.0104 | Cells_Transformed_fibroblasts |
| rs2664035 | SLAIN2 | 4 | 48220839 | A | G | 0.0104 | Cells_Transformed_fibroblasts |
| rs12936409 | GSDMA | 17 | 38043649 | T | C | 0.0118 | Cells_Transformed_fibroblasts |
| rs2596565 | Y_RNA | 6 | 31353329 | A | G | 0.0119 | Cells_Transformed_fibroblasts |
| rs998731 | RPSAP47 | 8 | 81095395 | T | C | 0.0123 | Cells_Transformed_fibroblasts |
| rs2596565 | HCP5 | 6 | 31353329 | A | G | 0.0126 | Cells_Transformed_fibroblasts |
| rs2596565 | PRR3 | 6 | 31353329 | A | G | 0.0135 | Cells_Transformed_fibroblasts |
| rs9603616 | FOXO1 | 13 | 40368069 | T | C | 0.0136 | Cells_Transformed_fibroblasts |
| rs2596565 | TCF19 | 6 | 31353329 | A | G | 0.0138 | Cells_Transformed_fibroblasts |
| rs660895 | PSMB9 | 6 | 32577380 | G | A | 0.0141 | Cells_Transformed_fibroblasts |
| rs11676922 | TXNDC9 | 2 | 100806940 | A | T | 0.0148 | Cells_Transformed_fibroblasts |
| rs1516971 | RP11-89M16.1 | 8 | 129542100 | C | T | 0.0157 | Cells_Transformed_fibroblasts |
| rs12936409 | GSDMB | 17 | 38043649 | T | C | 0.0159 | Cells_Transformed_fibroblasts |
| rs4272 | ERVW-1 | 7 | 92236829 | G | A | 0.0159 | Cells_Transformed_fibroblasts |
| rs34536443 | ZNF560 | 19 | 10463118 | C | G | 0.016 | Cells_Transformed_fibroblasts |
| rs2596565 | APOM | 6 | 31353329 | A | G | 0.0161 | Cells_Transformed_fibroblasts |
| rs968567 | ZBTB3 | 11 | 61595564 | T | C | 0.0168 | Cells_Transformed_fibroblasts |
| rs874040 | RBPJ | 4 | 26108197 | C | G | 0.0172 | Cells_Transformed_fibroblasts |
| rs2596565 | POU5F1 | 6 | 31353329 | A | G | 0.0183 | Cells_Transformed_fibroblasts |
| rs909685 | RP3-508I15.22 | 22 | 39747671 | A | T | 0.0184 | Cells_Transformed_fibroblasts |
| rs4409785 | AP001877.1 | 11 | 95311422 | C | T | 0.0188 | Cells_Transformed_fibroblasts |
| rs951005 | GALT | 9 | 34743681 | A | G | 0.019 | Cells_Transformed_fibroblasts |
| rs968567 | MYRF | 11 | 61595564 | T | C | 0.0207 | Cells_Transformed_fibroblasts |
| rs34695944 | USP34 | 2 | 61124850 | C | T | 0.0208 | Cells_Transformed_fibroblasts |
| rs951005 | STOML2 | 9 | 34743681 | A | G | 0.0209 | Cells_Transformed_fibroblasts |
| rs12936409 | MED24 | 17 | 38043649 | T | C | 0.0226 | Cells_Transformed_fibroblasts |
| rs2228145 | SHC1 | 1 | 154426970 | C | A | 0.0229 | Cells_Transformed_fibroblasts |
| rs11676922 | RP11-299H21.1 | 2 | 100806940 | A | T | 0.0229 | Cells_Transformed_fibroblasts |
| rs6920220 | PERP | 6 | 138006504 | A | G | 0.0234 | Cells_Transformed_fibroblasts |
| rs6715284 | FAM126B | 2 | 202154397 | G | C | 0.0238 | Cells_Transformed_fibroblasts |
| rs34536443 | PDE4A | 19 | 10463118 | C | G | 0.0242 | Cells_Transformed_fibroblasts |
| rs34536443 | RPS4XP22 | 19 | 10463118 | C | G | 0.0243 | Cells_Transformed_fibroblasts |
| rs968567 | DAGLA | 11 | 61595564 | T | C | 0.0246 | Cells_Transformed_fibroblasts |
| rs968567 | RAB3IL1 | 11 | 61595564 | T | C | 0.0247 | Cells_Transformed_fibroblasts |
| rs28411352 | C1orf122 | 1 | 38278579 | T | C | 0.025 | Cells_Transformed_fibroblasts |
| rs6715284 | CFLAR | 2 | 202154397 | G | C | 0.0251 | Cells_Transformed_fibroblasts |
| rs26232 | PAM | 5 | 102596720 | T | C | 0.0255 | Cells_Transformed_fibroblasts |
| rs951005 | RP11-331F9.3 | 9 | 34743681 | A | G | 0.0257 | Cells_Transformed_fibroblasts |
| rs10774624 | ADAM1B | 12 | 111833788 | A | G | 0.0262 | Cells_Transformed_fibroblasts |
| rs2596565 | PSORS1C2 | 6 | 31353329 | A | G | 0.0263 | Cells_Transformed_fibroblasts |
| rs4780401 | SNN | 16 | 11839326 | T | G | 0.0264 | Cells_Transformed_fibroblasts |
| rs28411352 | FHL3 | 1 | 38278579 | T | C | 0.0264 | Cells_Transformed_fibroblasts |
| rs2476601 | AP4B1 | 1 | 114377568 | G | A | 0.028 | Cells_Transformed_fibroblasts |
| rs2596565 | SKIV2L | 6 | 31353329 | A | G | 0.0295 | Cells_Transformed_fibroblasts |
| rs34695944 | RP11-493E12.1 | 2 | 61124850 | C | T | 0.0297 | Cells_Transformed_fibroblasts |
| rs12936409 | CSF3 | 17 | 38043649 | T | C | 0.0308 | Cells_Transformed_fibroblasts |
| rs8026898 | RP11-253M7.6 | 15 | 69991417 | A | G | 0.0315 | Cells_Transformed_fibroblasts |
| rs2596565 | ATF6B | 6 | 31353329 | A | G | 0.0315 | Cells_Transformed_fibroblasts |
| rs2596565 | CYP21A2 | 6 | 31353329 | A | G | 0.0333 | Cells_Transformed_fibroblasts |
| rs4409785 | MAML2 | 11 | 95311422 | C | T | 0.034 | Cells_Transformed_fibroblasts |
| rs9603616 | SLC25A15 | 13 | 40368069 | T | C | 0.0348 | Cells_Transformed_fibroblasts |
| rs951005 | FAM205A | 9 | 34743681 | A | G | 0.0364 | Cells_Transformed_fibroblasts |
| rs34695944 | NONOP2 | 2 | 61124850 | C | T | 0.0379 | Cells_Transformed_fibroblasts |
| rs9603616 | NHLRC3 | 13 | 40368069 | T | C | 0.038 | Cells_Transformed_fibroblasts |
| rs6715284 | NIF3L1 | 2 | 202154397 | G | C | 0.0391 | Cells_Transformed_fibroblasts |
| rs10774624 | ALDH2 | 12 | 111833788 | A | G | 0.0401 | Cells_Transformed_fibroblasts |
| rs951005 | RP11-133O22.6 | 9 | 34743681 | A | G | 0.0402 | Cells_Transformed_fibroblasts |
| rs706778 | NRBF2P5 | 10 | 6098949 | T | C | 0.0408 | Cells_Transformed_fibroblasts |
| rs2596565 | RANP1 | 6 | 31353329 | A | G | 0.0412 | Cells_Transformed_fibroblasts |
| rs883220 | MANEAL | 1 | 38616871 | A | C | 0.0413 | Cells_Transformed_fibroblasts |
| rs883220 | C1orf122 | 1 | 38616871 | A | C | 0.0414 | Cells_Transformed_fibroblasts |
| rs12936409 | PLXDC1 | 17 | 38043649 | T | C | 0.0416 | Cells_Transformed_fibroblasts |
| rs2596565 | RNF5 | 6 | 31353329 | A | G | 0.0417 | Cells_Transformed_fibroblasts |
| rs10488631 | RP11-274B21.2 | 7 | 128594183 | C | T | 0.0418 | Cells_Transformed_fibroblasts |
| rs2596565 | IER3 | 6 | 31353329 | A | G | 0.042 | Cells_Transformed_fibroblasts |
| rs34536443 | C19orf66 | 19 | 10463118 | C | G | 0.043 | Cells_Transformed_fibroblasts |
| rs998731 | TPD52 | 8 | 81095395 | T | C | 0.0444 | Cells_Transformed_fibroblasts |
| rs10488631 | IRF5 | 7 | 128594183 | C | T | 0.0446 | Cells_Transformed_fibroblasts |
| rs660895 | TAPBP | 6 | 32577380 | G | A | 0.046 | Cells_Transformed_fibroblasts |
| rs13330176 | RP11-118F19.1 | 16 | 86019087 | A | T | 0.0462 | Cells_Transformed_fibroblasts |
| rs660895 | C2 | 6 | 32577380 | G | A | 0.0463 | Cells_Transformed_fibroblasts |
| rs11676922 | RPL31 | 2 | 100806940 | A | T | 0.0466 | Cells_Transformed_fibroblasts |
| rs12936409 | CTD-2267D19.3 | 17 | 38043649 | T | C | 0.0471 | Cells_Transformed_fibroblasts |
| rs10488631 | RP11-274B21.4 | 7 | 128594183 | C | T | 0.0487 | Cells_Transformed_fibroblasts |
| rs6732565 | BUB1 | 2 | 111607832 | G | A | 0.049 | Cells_Transformed_fibroblasts |
| rs10774624 | TCTN1 | 12 | 111833788 | A | G | 0.0498 | Cells_Transformed_fibroblasts |
| rs968567 | FADS2 | 11 | 61595564 | T | C | 1.86E-71 | Whole_Blood |
| rs660895 | HLA-DQA2 | 6 | 32577380 | G | A | 9.33E-37 | Whole_Blood |
| rs12936409 | GSDMB | 17 | 38043649 | T | C | 3.13E-30 | Whole_Blood |
| rs12936409 | ORMDL3 | 17 | 38043649 | T | C | 2.39E-29 | Whole_Blood |
| rs909685 | SYNGR1 | 22 | 39747671 | A | T | 5.68E-21 | Whole_Blood |
| rs26232 | PPIP5K2 | 5 | 102596720 | T | C | 1.72E-19 | Whole_Blood |
| rs1893592 | UBASH3A | 21 | 43855067 | C | A | 8.07E-16 | Whole_Blood |
| rs3093023 | RNASET2 | 6 | 167534290 | A | G | 1.31E-15 | Whole_Blood |
| rs28411352 | INPP5B | 1 | 38278579 | T | C | 2.44E-14 | Whole_Blood |
| rs2843401 | MMEL1 | 1 | 2528133 | C | T | 2.45E-14 | Whole_Blood |
| rs660895 | HLA-DRB1 | 6 | 32577380 | G | A | 5.33E-14 | Whole_Blood |
| rs2596565 | C4B | 6 | 31353329 | A | G | 1.69E-13 | Whole_Blood |
| rs660895 | HLA-DQB2 | 6 | 32577380 | G | A | 7.88E-13 | Whole_Blood |
| rs660895 | HLA-DQB1 | 6 | 32577380 | G | A | 3.29E-12 | Whole_Blood |
| rs2596565 | C4A | 6 | 31353329 | A | G | 1.93E-10 | Whole_Blood |
| rs2596565 | LINC00243 | 6 | 31353329 | A | G | 1.04E-09 | Whole_Blood |
| rs3093023 | AL133458.1 | 6 | 167534290 | A | G | 1.16E-09 | Whole_Blood |
| rs4810485 | CD40 | 20 | 44747947 | G | T | 1.33E-09 | Whole_Blood |
| rs2596565 | CCHCR1 | 6 | 31353329 | A | G | 1.70E-09 | Whole_Blood |
| rs6715284 | PPIL3 | 2 | 202154397 | G | C | 1.07E-08 | Whole_Blood |
| rs2596565 | CYP21A1P | 6 | 31353329 | A | G | 4.63E-08 | Whole_Blood |
| rs660895 | HLA-DRB6 | 6 | 32577380 | G | A | 4.70E-08 | Whole_Blood |
| rs968567 | TMEM258 | 11 | 61595564 | T | C | 1.15E-07 | Whole_Blood |
| rs2228145 | IL6R | 1 | 154426970 | C | A | 3.20E-07 | Whole_Blood |
| rs2596565 | XXbac-BPG248L24.12 | 6 | 31353329 | A | G | 3.30E-07 | Whole_Blood |
| rs2596565 | CYP21A2 | 6 | 31353329 | A | G | 1.00E-06 | Whole_Blood |
| rs660895 | HLA-DQA1 | 6 | 32577380 | G | A | 1.30E-06 | Whole_Blood |
| rs660895 | HLA-DQB1-AS1 | 6 | 32577380 | G | A | 2.16E-06 | Whole_Blood |
| rs2843401 | FAM213B | 1 | 2528133 | C | T | 3.13E-06 | Whole_Blood |
| rs2596565 | HLA-S | 6 | 31353329 | A | G | 3.39E-06 | Whole_Blood |
| rs26232 | PAM | 5 | 102596720 | T | C | 3.98E-06 | Whole_Blood |
| rs660895 | LY6G5B | 6 | 32577380 | G | A | 1.41E-05 | Whole_Blood |
| rs3093023 | RPS6KA2 | 6 | 167534290 | A | G | 1.43E-05 | Whole_Blood |
| rs2596565 | FLOT1 | 6 | 31353329 | A | G | 2.43E-05 | Whole_Blood |
| rs2596565 | C2 | 6 | 31353329 | A | G | 5.94E-05 | Whole_Blood |
| rs28411352 | SF3A3 | 1 | 38278579 | T | C | 8.49E-05 | Whole_Blood |
| rs11676922 | AFF3 | 2 | 100806940 | A | T | 0.000109 | Whole_Blood |
| rs10774624 | ALDH2 | 12 | 111833788 | A | G | 0.000126 | Whole_Blood |
| rs660895 | TAP2 | 6 | 32577380 | G | A | 0.00019 | Whole_Blood |
| rs660895 | CYP21A1P | 6 | 32577380 | G | A | 0.00021 | Whole_Blood |
| rs34695944 | PUS10 | 2 | 61124850 | C | T | 0.000212 | Whole_Blood |
| rs6715284 | CASP8 | 2 | 202154397 | G | C | 0.000216 | Whole_Blood |
| rs2834512 | KCNE1 | 21 | 35911599 | A | G | 0.000221 | Whole_Blood |
| rs3093023 | CCR6 | 6 | 167534290 | A | G | 0.000224 | Whole_Blood |
| rs10488631 | TNPO3 | 7 | 128594183 | C | T | 0.000248 | Whole_Blood |
| rs2596565 | VARS2 | 6 | 31353329 | A | G | 0.000337 | Whole_Blood |
| rs2596565 | IER3 | 6 | 31353329 | A | G | 0.000502 | Whole_Blood |
| rs28411352 | FHL3 | 1 | 38278579 | T | C | 0.000586 | Whole_Blood |
| rs968567 | MYRF | 11 | 61595564 | T | C | 0.000605 | Whole_Blood |
| rs2843401 | RP3-395M20.8 | 1 | 2528133 | C | T | 0.000617 | Whole_Blood |
| rs10488631 | IRF5 | 7 | 128594183 | C | T | 0.000649 | Whole_Blood |
| rs6715284 | ALS2CR12 | 2 | 202154397 | G | C | 0.00102 | Whole_Blood |
| rs2228145 | RP11-350G8.7 | 1 | 154426970 | C | A | 0.00104 | Whole_Blood |
| rs660895 | FKBPL | 6 | 32577380 | G | A | 0.00125 | Whole_Blood |
| rs12936409 | RP11-94L15.2 | 17 | 38043649 | T | C | 0.00147 | Whole_Blood |
| rs6715284 | SUMO1 | 2 | 202154397 | G | C | 0.00212 | Whole_Blood |
| rs2596565 | HLA-C | 6 | 31353329 | A | G | 0.00219 | Whole_Blood |
| rs3093023 | FGFR1OP | 6 | 167534290 | A | G | 0.00248 | Whole_Blood |
| rs2596565 | CLIC1 | 6 | 31353329 | A | G | 0.0025 | Whole_Blood |
| rs660895 | HLA-DRB9 | 6 | 32577380 | G | A | 0.0027 | Whole_Blood |
| rs2596565 | POU5F1 | 6 | 31353329 | A | G | 0.00346 | Whole_Blood |
| rs9603616 | COG6 | 13 | 40368069 | T | C | 0.00389 | Whole_Blood |
| rs4810485 | AL031663.2 | 20 | 44747947 | G | T | 0.00397 | Whole_Blood |
| rs4780401 | TXNDC11 | 16 | 11839326 | T | G | 0.00399 | Whole_Blood |
| rs968567 | FADS1 | 11 | 61595564 | T | C | 0.00401 | Whole_Blood |
| rs1950897 | BANF1P1 | 14 | 68760141 | T | C | 0.00406 | Whole_Blood |
| rs12936409 | MSL1 | 17 | 38043649 | T | C | 0.0046 | Whole_Blood |
| rs34695944 | AHSA2 | 2 | 61124850 | C | T | 0.00486 | Whole_Blood |
| rs2843401 | RP11-345P4.9 | 1 | 2528133 | C | T | 0.00514 | Whole_Blood |
| rs3806624 | CMC1 | 3 | 27764623 | G | A | 0.00586 | Whole_Blood |
| rs9979383 | MORC3 | 21 | 36715761 | T | C | 0.00833 | Whole_Blood |
| rs660895 | BAG6 | 6 | 32577380 | G | A | 0.00849 | Whole_Blood |
| rs678347 | UBR5 | 8 | 102463602 | A | G | 0.00927 | Whole_Blood |
| rs909685 | APOBEC3C | 22 | 39747671 | A | T | 0.00949 | Whole_Blood |
| rs2596565 | PSORS1C3 | 6 | 31353329 | A | G | 0.0103 | Whole_Blood |
| rs10774624 | SH2B3 | 12 | 111833788 | A | G | 0.0103 | Whole_Blood |
| rs951005 | IL11RA | 9 | 34743681 | A | G | 0.0105 | Whole_Blood |
| rs660895 | PBX2 | 6 | 32577380 | G | A | 0.0109 | Whole_Blood |
| rs660895 | VARS | 6 | 32577380 | G | A | 0.0111 | Whole_Blood |
| rs8026898 | LINC00593 | 15 | 69991417 | A | G | 0.0116 | Whole_Blood |
| rs2596565 | XXbac-BPG252P9.10 | 6 | 31353329 | A | G | 0.0126 | Whole_Blood |
| rs660895 | SYNGAP1 | 6 | 32577380 | G | A | 0.0127 | Whole_Blood |
| rs874040 | TBC1D19 | 4 | 26108197 | C | G | 0.014 | Whole_Blood |
| rs10488631 | RP11-274B21.1 | 7 | 128594183 | C | T | 0.0141 | Whole_Blood |
| rs12936409 | CTD-2267D19.3 | 17 | 38043649 | T | C | 0.0141 | Whole_Blood |
| rs34536443 | TYK2 | 19 | 10463118 | C | G | 0.0144 | Whole_Blood |
| rs909685 | UQCRFS1P1 | 22 | 39747671 | A | T | 0.0147 | Whole_Blood |
| rs2596565 | NEU1 | 6 | 31353329 | A | G | 0.0148 | Whole_Blood |
| rs2834512 | RCAN1 | 21 | 35911599 | A | G | 0.0152 | Whole_Blood |
| rs2228145 | S100A16 | 1 | 154426970 | C | A | 0.0152 | Whole_Blood |
| rs2228145 | S100A3 | 1 | 154426970 | C | A | 0.0159 | Whole_Blood |
| rs13330176 | MTHFSD | 16 | 86019087 | A | T | 0.016 | Whole_Blood |
| rs968567 | PRPF19 | 11 | 61595564 | T | C | 0.0163 | Whole_Blood |
| rs4780401 | ZC3H7A | 16 | 11839326 | T | G | 0.018 | Whole_Blood |
| rs3093023 | RP1-167A14.2 | 6 | 167534290 | A | G | 0.0193 | Whole_Blood |
| rs951005 | UBAP1 | 9 | 34743681 | A | G | 0.0198 | Whole_Blood |
| rs2275806 | ATP5C1 | 10 | 8095340 | A | G | 0.0199 | Whole_Blood |
| rs951005 | DCTN3 | 9 | 34743681 | A | G | 0.0216 | Whole_Blood |
| rs2596565 | AGER | 6 | 31353329 | A | G | 0.0224 | Whole_Blood |
| rs12936409 | GSDMA | 17 | 38043649 | T | C | 0.0226 | Whole_Blood |
| rs660895 | HLA-DRA | 6 | 32577380 | G | A | 0.0227 | Whole_Blood |
| rs2228145 | NPR1 | 1 | 154426970 | C | A | 0.0231 | Whole_Blood |
| rs660895 | HLA-DMA | 6 | 32577380 | G | A | 0.0235 | Whole_Blood |
| rs595158 | TMEM258 | 11 | 60909581 | A | C | 0.0236 | Whole_Blood |
| rs595158 | CYB561A3 | 11 | 60909581 | A | C | 0.0237 | Whole_Blood |
| rs951005 | RP11-195F19.5 | 9 | 34743681 | A | G | 0.0238 | Whole_Blood |
| rs2228145 | UBE2Q1 | 1 | 154426970 | C | A | 0.0245 | Whole_Blood |
| rs2596565 | C6orf25 | 6 | 31353329 | A | G | 0.0248 | Whole_Blood |
| rs34536443 | RAVER1 | 19 | 10463118 | C | G | 0.025 | Whole_Blood |
| rs2596565 | XXbac-BPG299F13.17 | 6 | 31353329 | A | G | 0.0259 | Whole_Blood |
| rs874040 | SEL1L3 | 4 | 26108197 | C | G | 0.0262 | Whole_Blood |
| rs660895 | CSNK2B | 6 | 32577380 | G | A | 0.0263 | Whole_Blood |
| rs10488631 | CICP14 | 7 | 128594183 | C | T | 0.0263 | Whole_Blood |
| rs951005 | RUSC2 | 9 | 34743681 | A | G | 0.0265 | Whole_Blood |
| rs4810485 | SDC4 | 20 | 44747947 | G | T | 0.0271 | Whole_Blood |
| rs34536443 | KEAP1 | 19 | 10463118 | C | G | 0.0272 | Whole_Blood |
| rs34536443 | SLC44A2 | 19 | 10463118 | C | G | 0.0278 | Whole_Blood |
| rs909685 | DDX17 | 22 | 39747671 | A | T | 0.0285 | Whole_Blood |
| rs2228145 | GBA | 1 | 154426970 | C | A | 0.0291 | Whole_Blood |
| rs951005 | RP11-176F3.7 | 9 | 34743681 | A | G | 0.0293 | Whole_Blood |
| rs4780401 | SNX29 | 16 | 11839326 | T | G | 0.0297 | Whole_Blood |
| rs2596565 | PRR3 | 6 | 31353329 | A | G | 0.03 | Whole_Blood |
| rs4272 | RBM48 | 7 | 92236829 | G | A | 0.0311 | Whole_Blood |
| rs10774624 | GPN3 | 12 | 111833788 | A | G | 0.0317 | Whole_Blood |
| rs2228145 | S100A1 | 1 | 154426970 | C | A | 0.0321 | Whole_Blood |
| rs10774624 | RP3-462E2.3 | 12 | 111833788 | A | G | 0.0351 | Whole_Blood |
| rs28411352 | MEAF6 | 1 | 38278579 | T | C | 0.0351 | Whole_Blood |
| rs4452313 | GALNT15 | 3 | 17047032 | T | A | 0.0359 | Whole_Blood |
| rs2476601 | AP4B1 | 1 | 114377568 | G | A | 0.0359 | Whole_Blood |
| rs10774624 | FAM109A | 12 | 111833788 | A | G | 0.0365 | Whole_Blood |
| rs6920220 | PEX7 | 6 | 138006504 | A | G | 0.0378 | Whole_Blood |
| rs4780401 | SNN | 16 | 11839326 | T | G | 0.0378 | Whole_Blood |
| rs660895 | ABHD16A | 6 | 32577380 | G | A | 0.041 | Whole_Blood |
| rs951005 | RMRP | 9 | 34743681 | A | G | 0.0412 | Whole_Blood |
| rs968567 | TAF6L | 11 | 61595564 | T | C | 0.0417 | Whole_Blood |
| rs951005 | RP11-331F9.3 | 9 | 34743681 | A | G | 0.0421 | Whole_Blood |
| rs706778 | NRBF2P5 | 10 | 6098949 | T | C | 0.0423 | Whole_Blood |
| rs968567 | PGA3 | 11 | 61595564 | T | C | 0.0429 | Whole_Blood |
| rs660895 | TAPSAR1 | 6 | 32577380 | G | A | 0.0431 | Whole_Blood |
| rs595158 | RN7SL23P | 11 | 60909581 | A | C | 0.0435 | Whole_Blood |
| rs2834512 | CMP21-97G8.1 | 21 | 35911599 | A | G | 0.0437 | Whole_Blood |
| rs874040 | SEPSECS | 4 | 26108197 | C | G | 0.0451 | Whole_Blood |
| rs2596565 | LST1 | 6 | 31353329 | A | G | 0.0456 | Whole_Blood |
| rs909685 | RP4-742C19.12 | 22 | 39747671 | A | T | 0.0457 | Whole_Blood |
| rs874040 | SMIM20 | 4 | 26108197 | C | G | 0.0466 | Whole_Blood |
| rs28411352 | C1orf122 | 1 | 38278579 | T | C | 0.048 | Whole_Blood |
| rs34536443 | C3P1 | 19 | 10463118 | C | G | 0.0493 | Whole_Blood |
